# Detection and Identification of Novel Intracellular Bacteria Hosted in Strains CBS 648.67 and CFCC 80795 of Biocontrol Fungi *Metarhizium*

**DOI:** 10.1264/jsme2.ME21059

**Published:** 2022-05-24

**Authors:** Yue Ying, Chenglin Liu, Ran He, Ruizhen Wang, Liangjian Qu

**Affiliations:** 1 Key Laboratory of Forest Protection of National Forestry and Grassland Administration, Ecology and Nature Conservation Institute, Chinese Academy of Forestry, Beijing 100091, China; 2 Beijing Floriculture Engineering Technology Research Centre, Beijing Botanical Garden, Beijing 100093, China

**Keywords:** endosymbiosis, endobacteria, *Metarhizium bibionidarum*, *Metarhizium anisopliae*

## Abstract

“Endosymbiosis” is a cohesive form of a symbiotic association. Endobacteria exist in many fungi and play important roles in fungal host biology. *Metarhizium* spp. are important entomopathogenic fungi for insect pest control. In the present study, we performed comprehensive ana­lyses of strains of *Metarhizium bibionidarum* and *M. anisopliae* using PCR, phylogenetics, and fluorescent electron microscopy to identify endobacteria within hyphae and conidia. The results of the phylogenetic ana­lysis based on 16S rRNA gene sequences indicated that these endobacteria were the most closely related to *Pelomonas puraquae* and affiliated with *Betaproteobacteria*. Ultrastructural observations indicated that endobacteria were coccoid and less than 500‍ ‍nm in diameter. The basic characteristics of endobacteria in *M. bibionidarum* and *M. anisopliae* were elucidated, and biological questions were raised regarding their biological functions in the *Metarhizium* hosts.

In natural ecosystems, symbiosis is an important association between animals, plants, fungi, and prokaryotes (Frey-Klett *et al.*, 2007; Kikuchi, 2009; Giordano *et al.*, 2013). Symbionts often play critical roles in development, physiology, behavior, defense, and a number of other traits in a host (Selosse *et al.*, 2004; Leung and Poulin, 2008; McFall-Ngai *et al.*, 2013; Gerardo and Parker, 2014). Symbiotic relationships in which both species in the association benefit are mutualistic (Das and Varma, 2009). Mutualistic relationships between bacteria and hosts (animals, plants, and fungi) are very common (Fraune and Bosch, 2010; Bonfante and Desiro, 2017; Zipfel and Oldroyd, 2017). In mutualism, hosts often provide bacteria with a living space and the nutrients needed for growth (Anca *et al.*, 2009; Schüßler, 2012; Bonfante *et al.*, 2019), while bacteria play important roles in host biology, including aspects of host reproduction (Partida-Martinez *et al.*, 2007; Mondo *et al.*, 2017), growth (Guo *et al.*, 2017; Shaffer *et al.*, 2017; Desiro *et al.*, 2018), energy dynamics (Vannini *et al.*, 2016; Salvioli *et al.*, 2017), primary metabolism (Vannini *et al.*, 2016; Li *et al.*, 2017; Uehling *et al.*, 2017), and secondary metabolism (Rohm *et al.*, 2010; Hoffman *et al.*, 2013; Deveau *et al.*, 2018). In addition to living on the body surface or within the tissues of the host in the extracellular space, bacteria may also inhabit the cytoplasm, forming the most intimate interaction, called “endosymbiosis”. These bacteria are termed cytoplasmic endobacteria (Bonfante *et al.*, 2019).

Endosymbiosis between bacteria and plants/animals has been extensively examined for many years (Haney and Ausubel, 2015; Santos *et al.*, 2018; Skiada *et al.*, 2019); however, the study of bacterial-fungal interactions has only emerged in recent years (Bonfante and Desiro, 2017). Several fungal lineages possess endobacteria, including *Mucoromycota*, *Glomeromycota*, *Ascomycota*, and *Basidiomycota* (Hoffman and Arnold, 2010; Deveau *et al.*, 2018; Liu *et al.*, 2019). Known endobacteria include *Alphaproteobacteria*, *Betaproteobacteria*, *Firmicutes*, *Actinobacteria*, and *Gammaproteobacteria*, and the functions of these endobacteria in their hosts have been preliminarily investigated and speculated (Rohm *et al.*, 2010; Liu *et al.*, 2019).

Recent studies on fungal endobacteria have mainly focused on the endobacteria of fungi with plant hosts (Bonfante *et al.*, 2019; Liu *et al.*, 2019), while limited information is currently available on the endobacteria of entomopathogenic fungi. *Metarhizium* spp. are natural enemies of a wide range of insects and arachnids, and, thus, they are being used as environmentally friendly alternatives to chemical insecticides (Gao *et al.*, 2011). *Metarhizium* spp. are one of the most widely used fungi and mycoinsecticides worldwide (Zimmermann, 2007), with *Metarhizium acridum* being used to manage and prevent infestations by the superfamily Acridoidea (Gao *et al.*, 2011). In addition, *M. anisopliae* is effective in the control of malaria-vectoring mosquitoes (Culicidae, Diptera) as well as scarabs and aphids (Scholte *et al.*, 2005; Kanzok and Jacobs-Lorena, 2006; Schrank and Vainstein, 2010). The phylogeny (Driver *et al.*, 2000; Bischoff *et al.*, 2009), infection mechanisms, virulence (Schrank and Vainstein, 2010), safety, environmental stability (Zimmermann, 2007), strain improvements (Roberts and Leger, 2004), and other aspects of *Metarhizium* spp. have been extensively examined; however, the endobacteria of *Metarhizium* spp. remain unknown.

Through preliminary bacterial sequence amplification and fluorescent nucleic acid staining, we detected endosymbiotic bacteria in *Metarhizium* spp. Endobacteria may be important to the biology and ecology of *Metarhizium* spp. To obtain a more detailed understanding of the presence and spatial distribution of endobacteria in *Metarhizium* spp., we investigated the domestic and foreign strains, CBS 648.67 (Nishi *et al.*, 2017) and CFCC 80795, respectively. CBS 648.67 and CFCC 80795 belong to the two most common species of *Metarhizium* spp., *M. bibionidarum* (a member of the *M. flavoviride* species complex) and *M. anisopliae*, respectively. These strains of the genus are also commonly used in *Metarhizium* insecticides (Milner, 2000; Nong *et al.*, 2015; Brunner-Mendoza *et al.*, 2018; Mascarin *et al.*, 2019). Previous pathogenicity experiments against *Monochamus alternatus* adults revealed the good insecticidal efficacies of these two strains (Y. Ying *et al.*, unpublished). In the present study, the ultrastructural morphology and localization of endobacteria in hyphae and conidia were confirmed using a combination of fluorescence *in situ* hybridization (FISH), transmission electron microscopy (TEM), and scanning electron microscopy (SEM). The phylogeny of endosymbionts was revealed by sequencing and phylogenetic ana­lyses. The results obtained herein revealed the presence of endobacteria in *Metarhizium* spp., and provide a new perspective for understanding the complex interactions between *Metarhizium* spp., endobacteria, and host insects.

## Materials and Methods

### Fungal strain

The biological control fungal strain CBS 648.67 (*M. bibionidarum*) was purchased from Centraalbureau voor Schimmelcultures (CBS), and strain CFCC 80795 (*M. anisopliae*) was provided by the China Forestry Culture Collection Center.

### Fungal culture conditions

The mycelia or conidia of fungal cultures were used to inoculate solid potato dextrose agar (PDA; Becton, Dickinson and Co.) medium or liquid potato sucrose (L^–1^: 200‍ ‍g fresh potato and 20‍ ‍g sucrose [Sinopharm Chemical Reagent]) medium at 25°C.

### Isolation and culture of endobacteria

The mycelia of 7-day-old CBS 648.67 and CFCC 80795 grown in liquid-submerged cultures were filtered and cryo-ground (0–4°C) using RETSCH’s Mixer Mill MM 400. After grinding, products were mixed with 0.25 M sucrose solution and centrifuged at different rotational speeds to obtain isolated bacteria. Supernatants were collected and filtered through cellulose nitrate (Cellulose Nitrate [Mixed Cellulose Ester] Membrane Filters, 5‍ ‍μm, Sartorius) to obtain suspensions containing endobacteria. They were then centrifuged at 13,000×*g* at 4°C for 20‍ ‍min. The supernatants were gently removed to avoid losing endobacteria, which were resuspended in 0.25 M sucrose solution. Suspensions were plated onto agar plates and incubated at 25°C.

Each bacterial suspension was spread onto two media (200‍ ‍μL of suspensions per plate): LB agar (L^–1^: 10‍ ‍g tryptone [Becton, Dickinson and Co.], 10‍ ‍g sodium chloride [Sinopharm Chemical Reagent], 5‍ ‍g yeast extract [Becton, Dickinson and Co.], and 1.5% [w/v] agar [Sinopharm Chemical Reagent]) and R2A agar (Becton, Dickinson and Co.), and then incubated at 25°C for 2‍ ‍weeks. R2A agar is suitable for *Pelomonas* growth according to Gomila *et al.* (2007), and LB agar is commonly used as a bacterial medium. Therefore, these two media were selected for the cultivation of endobacteria. Ten plates were used to observe the growth of endobacteria.

### DNA extraction, amplification, and clone library ana­lysis

Fungal hyphae were filtered under sterile conditions and ground to a fine powder in liquid nitrogen. DNA was isolated using the TaKaRa MiniBEST Plant Genomic DNA Extraction Kit (TaKaRa Bio) in accordance with the instruction manual in a sterile environment. Endobacterial 16S rRNA genes and *mreB* cytoskeletal protein-encoding genes were amplified using the universal bacterial primer pairs 27f (5′-GAGAGTTTGATCCTGGCTCAG-3′)/1492r (5′-TACGGYTACCTTGTTACGACTT-3′) (Weisburg *et al.*, 1991) and mreB-1 (5′-GATGAATTCAGTCCACATCGCAATCTG-3′)/mreB-2 (5′-CCACTCGAGTACCAAATTCCCTTTACG-3′) (Gaballah *et al.*, 2011), and ten pairs of primers (Table S1) were designed using Primer3Plus (Untergasser *et al.*, 2007) based on the approximately 1,043-bp *mreB* gene of *Pelomonas* spp. in NCBI. The PCR program consisted of 94°C for the initial 2‍ ‍min, 30 cycles at 95°C with 1‍ ‍min of denaturing, at 52°C with 90‍ ‍s of annealing, and at 68°C with 2‍ ‍min of extension, followed by a final extension for 10‍ ‍min. The primers 19F (5′-GCIWTYTAYGGIAARGGIGG-3′) and 407R (5′-AAICCRCCRCAIACIACRTC-3′) were used to amplify the approximately 388-bp *nifH* gene encoding the dinitrogenase reductase iron protein. Reaction conditions were an initial step at 94°C for 5‍ ‍min, 40 cycles at 94°C for 30‍ ‍s, at 50°C for 1‍ ‍min, and at 72°C for 30‍ ‍s, followed by a final step at 72°C for 10‍ ‍min (Ueda *et al.*, 1995). The primers *hox*F1 (5′-GAYCCNRTNACNMGNATHGARGGNCA-3′) and *hox*R1 (5′-ACRTGNRYBSVRCANSCVRDVMANGGRTC-3′) were used for the amplification of the approximately 1,800-bp hydrogenase gene *hoxG* (Kortlüke *et al.*, 1992). The PCR program used was an initial denaturation step at 94°C for 5‍ ‍min, 5 cycles at 94°C for 1‍ ‍min, at 65°C for 1‍ ‍min, and at 72°C for 105‍ ‍s, 20 cycles at 94°C for 1‍ ‍min, at 65°C for 1‍ ‍min (a decrease of 0.5°C per cycle), and at 72°C for 105 s; 15 cycles at 94°C for 1‍ ‍min, at 55°C for 1‍ ‍min, and at 72°C for 105 s; and finally an extension at 72°C for 10‍ ‍min (Gomila *et al.*, 2007).

PCR amplification was accomplished using the Gene Amp PCR System 2400 (PerkinElmer). The 50-μL PCR reaction volume contained 0.25‍ ‍μL DNA Polymerase LA Taq (TaKaRa Bio.) (5‍ ‍U μL^–1^), 5‍ ‍μL 10× PCR buffer (Mg^2+^ Plus), 4‍ ‍μL dNTP mixture (2.5‍ ‍mM), 1‍ ‍μL of each primer (20‍ ‍μM), and 2.5‍ ‍ng DNA.

PCR products from bacterial amplification were cloned using the pGEM-t Easy Vector System (Promega) and then transformed into DH5α Chemically Competent *Escherichia coli* (Invitrogen). Ten colonies harboring bacterial fragments were sequenced and analyzed. Colonies were screened for insert lengths. Bacterial clones were sequenced on an ABI 3730xl automated sequencer (Applied Biosystems).

Sequence data were deposited in the NCBI database under the accession numbers MZ686431, OM530146, and MZ822939. MZ686431 and OM530146 were the accession numbers of the 16S rRNA gene sequences of CBS 648.67 and CFCC80795, respectively. MZ822939 was the sequence amplified by the primer pairs mreB-1/mreB-2.

### Phylogenetic ana­lysis

The 16S rRNA gene sequences of endobacteria in the two strains, sharing >98% identities with the BLAST hits of target sequences from GenBank (30 sequences), were aligned using MUSCLE version 3.8.31 (Edgar, 2004). We also included the closely related genus *Thiomonas*, with *Thiomonas thermosulfata* strain ATCC 51520 serving as the outgroup. JModelTest was used to choose the substitution model for the phylogeny (Guindon and Gascuel, 2003; Darriba *et al.*, 2012).

Phylogenetic ana­lyses were performed using Bayesian and Maximum-likelihood methods. Bayesian tree sampling was conducted using the MrBayes 3.2.7 program (Ronquist *et al.*, 2012). We employed the Tamura-Nei model of nucleotide evolution, including estimations of invariant sites and the assumption of a discrete gamma distribution (TrN+I+G). The inference consisted of 1,998,000 generations (stopped automatically due to the convergence of all parameters) with sampling every 100 generations. The first 25% of samples were discarded as burn-in (Ronquist *et al.*, 2012). Maximum-likelihood ana­lyses were performed using the program MEGA version 7.0 (Kumar *et al.*, 2016), and maximum-likelihood trees were constructed using the TrN+I+G model and 1,000 bootstraps replicates (Edgar, 2004; Kumar *et al.*, 2016). Tree results were viewed and edited using the program FigTree v1.4.4 (http://tree.bio.ed.ac.uk/software/figtree/).

### Localization of endobacteria by fluorescence

#### Fluorescent nucleic acid stain

The SYTO 9 green-fluorescent nucleic acid stain (Molecular Probes) and microscopic examinations were used to establish whether the small intracellular bodies observed within hyphae were bacteria. In accordance with the manufacturer’s instructions, 1.5‍ ‍μL SYTO 9 was added per mL fungal suspension. The suspension and dye were mixed thoroughly and incubated at room temperature in the dark for 20‍ ‍min. Stained hyphae and conidia were viewed using a confocal laser scanning microscope (Leica TCS SP5, Leica). The hyphae and conidia of four cultures of each strain were observed.

#### FISH

One-week-old fungal cultures grown on PDA were harvested and fixed in 50% ethanol with phosphate-buffered saline (pH 7.0) at 4°C for 24 h. Cultures were then washed three times in 1× PBS and treated with Viscozyme^®^ L (Sigma-Aldrich) at 37°C for 2 h and 0.2 M HCl at 25°C for 0.5 h to increase cell wall permeability. Fixed fungal material was dehydrated in an increasing ethanol series (50, 80, and 100% [v/v]) for 3‍ ‍min each. Endobacteria suspensions (see *Isolation and culture of endobacteria*) were centrifuged at 12,000×*g* for 5‍ ‍min. Supernatants were removed and centrifugation was repeated after the addition of distilled water for washing. Distilled water was added to resuspend pellets. FISH was performed as described by Fuchs *et al.* (2007) and Sharma *et al.* (2008).

Between 2 and 20‍ ‍μL of the treated cell suspension (depending on cell density) was spotted onto the wells of “PTFE” Printed Slides (Electron Microscopy Sciences), air-dried, and then dehydrated for 3‍ ‍min each in 50, 80, and 100% ethanol. Hybridization in 10‍ ‍μL of the hybridization mix (including 10‍ ‍μM of each probe) per well was performed at 46°C for 3 h, followed by a stringent washing step at 48°C for 10–15‍ ‍min. All steps in FISH with fungal material were conducted in 50-mL polyethylene tubes (sterile). Samples were incubated with 10‍ ‍μL of 1‍ ‍μg mL^–1^ 4′,6-diamidine-2′-phenylindole dihydrochloride (DAPI; Acmec) for 3‍ ‍min to stain fungal DNA, and slides were then rinsed with distilled H_2_O. Before observations, slides were mounted in VECTASHIELD^®^ Mounting Media H-1000 (Vector Laboratories) and viewed using confocal laser scanning microscopy (Zeiss LSM980 with Elyra7, Zeiss).

Oligonucleotide probes were designed using Primer3Plus (probe 16S: 5′-TACCCCACCAACTACCTA-3′, in accordance with the sequence amplified by the primer pair 27f/1492r, and probe M [5′-AAACACCCACAATAGCCTGC-3′)] based on the sequence amplified by the primer pair mreB-1/mreB-2). The oligonucleotide probe EUB338 mix included EUB338, EUB338 II, and EUB338 III for bacteria (Daims *et al.*, 1999). Probe 16S and the EUB338 mix were fluorescently labeled with 6-FAM at the 5′ ends, and probe M was fluorescently labeled with Cy5 at the 5′ end. The probes were synthesized by Sangon Biotech and diluted with sterile water in accordance with the manufacturer’s instructions.

Fungal cultures of each strain were used four times to perform the preceding operations, and each culture was sampled from at least three wells for observations.

### Ultrastructural morphology of bacterial symbionts

#### TEM

Hyphae and conidia from PDA cultures grown for 7 days were used for TEM observations. Samples were frozen in a high-pressure freezer (Leica EM HPM100; Leica). Fast-frozen samples were then immersed into a freezing tube containing osmic acid (2%) and placed into the freeze substitution device (Leica EM AFS; Leica) at –90°C for 3 days. They were then slowly warmed to 4°C. Following freezing substitution, samples were rinsed four times with 100% acetone at room temperature. Dehydrated specimens were slowly infiltrated with SPI Pon 812 resin by placing them in mixtures of acetone and resin of different grades (25, 50, 75, and 100% [v/v]). The liquid resin was then polymerized at 60°C for 48 h. Ultrathin sections were cut using an ultramicrotome (Leica EM UC6; Leica) equipped with a diamond knife and placed on a TEM grid. The emulsion was observed using TEM (FEI Tacnai Spirit) at 100 kV. Four cultures of each strain were observed.

#### SEM

Isolated bacteria (see *Isolation and culture of endobacteria*) were fixed in 1.5‍ ‍mL of 2.5% (v/v) glutaraldehyde prepared in 0.1-M PBS buffer (pH 7.0) and incubated at 4°C overnight. After the fixation step, each sample was rinsed three times in 0.1 M PBS buffer (pH 7.0) on ice. Specimens were then dehydrated. The dehydration process included the immersion of specimens in 50% (v/v) ethanol at 4°C for 10‍ ‍min, 70% (v/v) ethanol for 10‍ ‍min, 80% (v/v) ethanol for 10‍ ‍min, 90% (v/v) ethanol for 15‍ ‍min, and 100% absolute ethyl ethanol (dried with CaCl_2_) twice for 20‍ ‍min. Specimens were then critical-point dried with CO_2_ as a transitional fluid and finally sputter coated with gold-palladium using an ion-sputtering device (JFC-1100; JEOL). Each sample was observed and photographed using a scanning electron microscope (S-4800; Hitachi). Four cultures of each strain were observed.

## Results

### SYTO 9 staining of fungal conidia

**Fig. 1.**
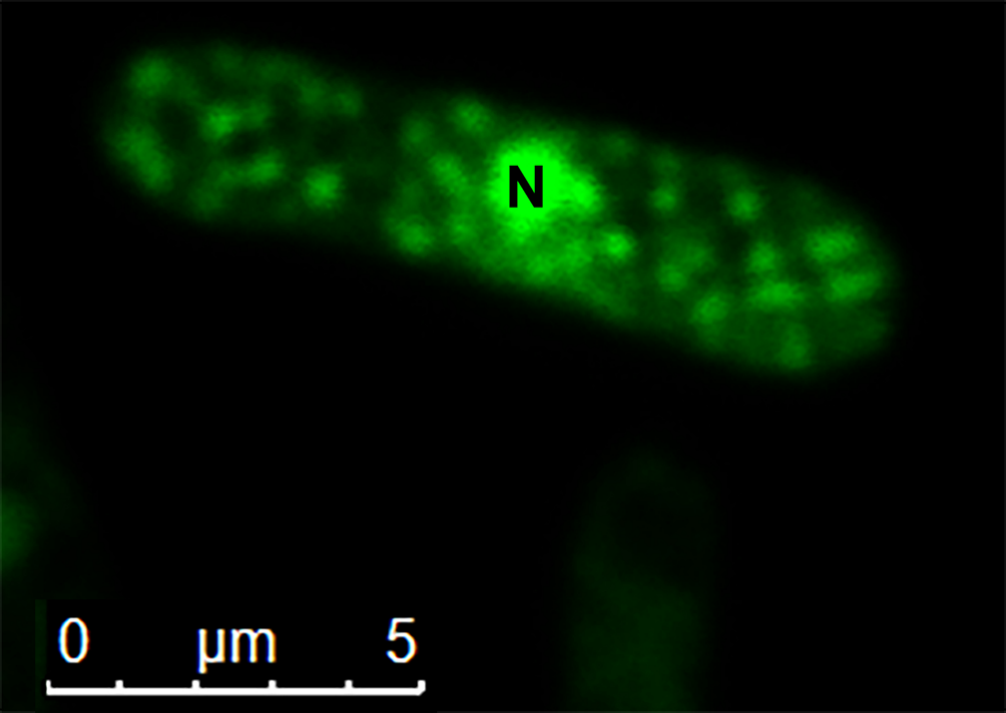
Nucleic acid staining of a conidium of *Metarhizium bibionidarum* CBS 648.67 using SYTO 9. Endobacteria in conidia are observed as bright green fluorescent spots; N, nucleus.

SYTO 9-stained DNA fluoresces bright green under blue excitation. The axenic conidia of *M. anisopliae* were stained with SYTO 9 dye. Many green fluorescent signal spots were observed in the cytoplasm of conidia in addition to fungal nuclei (Fig. 1). These green fluorescent dots indicated endobacteria or other DNA-containing organelles in fungal conidia.

### Isolation and cultivation of endobacteria

The endobacteria isolated from CBS 648.67 and CFCC 80795 were cultured in two culture media at 25°C for 2‍ ‍weeks under sterile conditions to investigate their free-living capacities. Endobacterial growth was not observed in any of the media tested.

### Endobacterial identification and phylogenetic tree construction

**Fig. 2.**
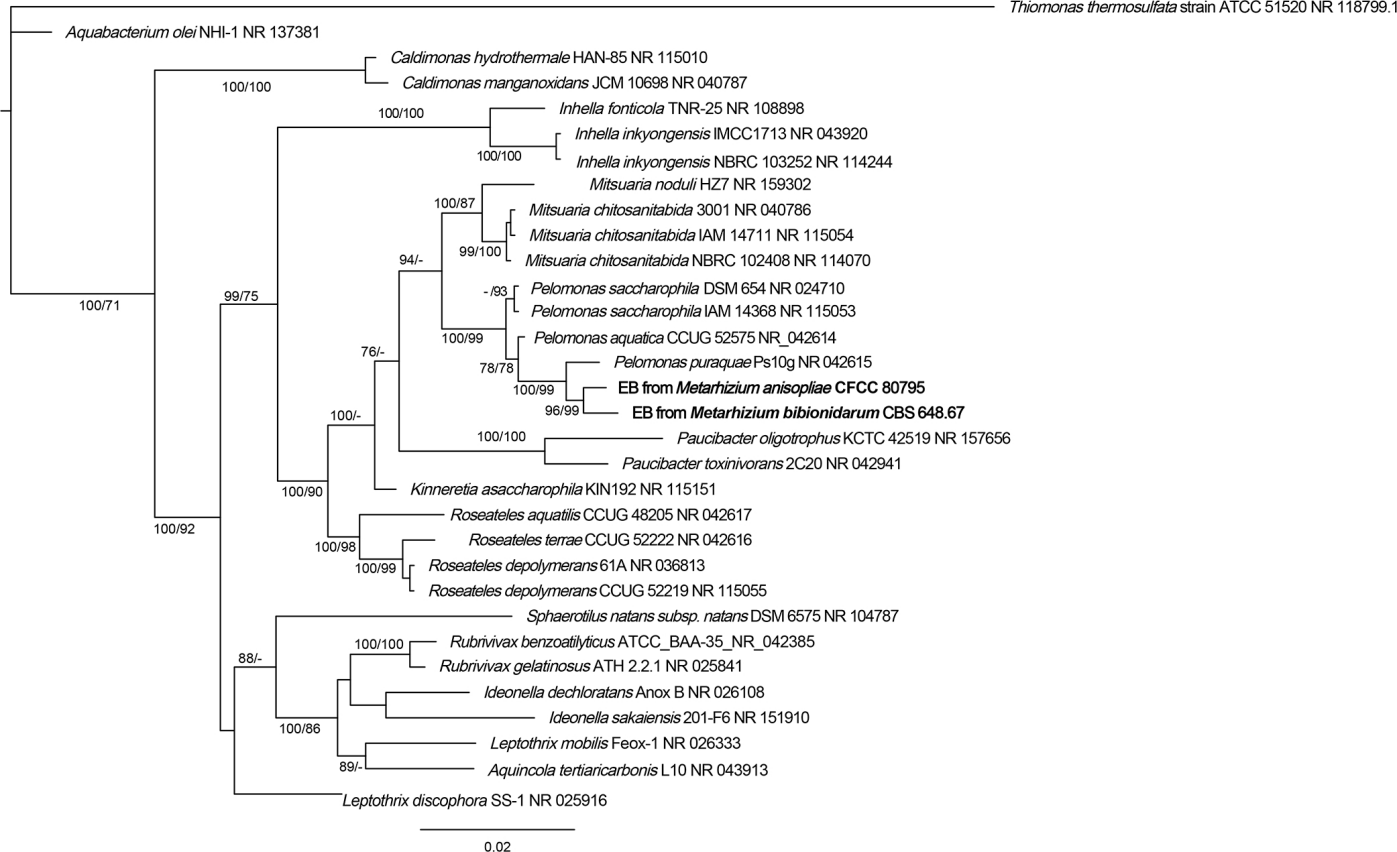
Phylogenetic placement of endobacterial 16S rRNA gene sequences. The DNA sequences retrieved in this study are shown in bold. Both samples are located inside the *Pelomonas* clade, close to *Pelomonas* *puraquae*. Supported values are from Bayesian/Maximum-likelihood methods. Bayesian and maximum-likelihood ana­lyses were performed with TrN+I+G nucleotide substitution models for 16S rRNA regions. Dashes instead of numbers indicate that the topology has supported values <70%.

Bacterial 16S rRNA gene sequences were amplified by PCR using the universal bacterial primer pair 27f/1492r and sequenced to detect the presence of endosymbionts. Phylogenetic affiliations were then analyzed. PCR experiments revealed that endobacteria were always present in the surface-sterilized hyphae and conidia of the two strains (Fig. 2). Database searches using these sequences as queries indicated shared highest identity levels with *Pelomonas* spp. sequences in the NCBI database. Furthermore, a phylogenetic ana­lysis based on 16S rDNA revealed that the endobacterial sequences from the two fungal strains of different geographic origins all clustered in a well-supported clade that was the most closely related to *Pelomonas puraquae* and affiliated with *Betaproteobacteria* (Fig. 2). Since endobacteria were broadly present inside two different *Metarhizium* strains, their occurrence did not appear to be an accidental or sporadic phenomenon.

Nitrogenase (*nifH*) and hydrogenase (*hoxG*) genes was amplified using the primer pairs 19F/407R and *hox*F1/*hox*R1. Gel images of the PCR products (Fig. S1) generated using the two primer pairs showed multiple bands; however, multi-band clones were not related to the *nifH* or *hoxG* genes of *Pelomonas* spp. Therefore, the *nifH* and *hoxG* genes of the endobacteria associated with *Pelomonas* spp. were absent.

Eleven primer pairs were used for the amplification of the *mreB* gene. A 1,471-bp sequence was obtained using the primer pair mreB-1/mreB-2. Its length and alignment revealed that it was not the *mreB* gene of *P. puraquae*, but a sequence with an unknown function. The sequence was highly similar to one from *M. brunneum* strain CP058938.1 (query cover 98%, identity 96.14%) according to the BLAST results in the NCBI database. These results also showed that none of the sequences amplified using the ten designed primer pairs were related to the *mreB* gene of *P. puraquae*.

### FISH

**Fig. 3.**
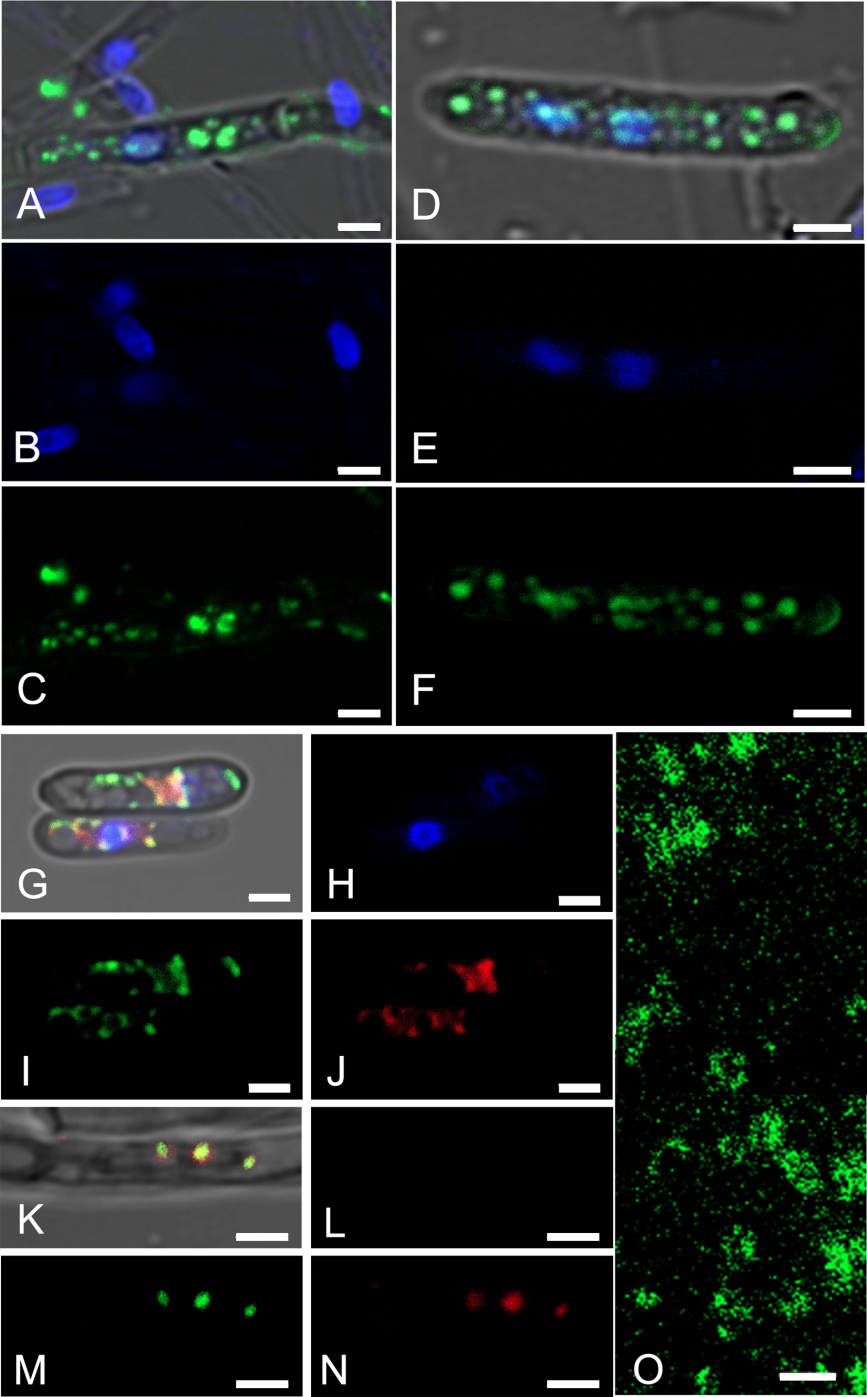
Detection of endobacteria by fluorescence *in situ* hybridization (FISH). FISH was performed with EUB338 mix (green), probe 16S (green), probe M (red), and DAPI (blue). (A, B, C, D, E, and F) Double labeling with the universal bacterial probe EUB338 mix and DAPI. (G, H, I, J, K, L, M, and N) Triple labeling with probe 16S, probe M, and DAPI. (O) Isolated endobacteria with the probe EUB338 mix. (A, D, G, K) Superimposed images. (B, E, H, L) Blue channel. (C, F, I, M, O) Green channel. (J, N) Red channel. Hyphae (A, B, C, K, L, M, and N), isolated endobacteria (O), and conidia (D, E, G, H, I, and J). Nuclei are observed as blue fluorescence spots. Scale bars, 2‍ ‍μm.

To confirm that bacteria were from inside the fungi and to identify the intracytoplasmic localization of bacterial cells, conidia and hyphae were stained and detected using FISH and confocal laser scanning microscopy. Endobacteria presented as coccoid fluorescent spots in the cytoplasm of conidia and hyphae using the EUB338 mix, probe 16S, and probe M (Fig. 3A, B, C, D, E, F, G, H, I, J, K, L, M, and N). Isolated bacteria also presented as coccoid fluorescent spots (Fig. 3O). FISH indicated that the signals stained by 6-FAM and Cy5 were endobacteria. Z-stacked images (Fig. S2, 3, 4, and 5) and time series images (Fig. S6) showed fluorescence signals in conidia and hyphae.

### Ultrastructural morphology of endobacteria

**Fig. 4.**
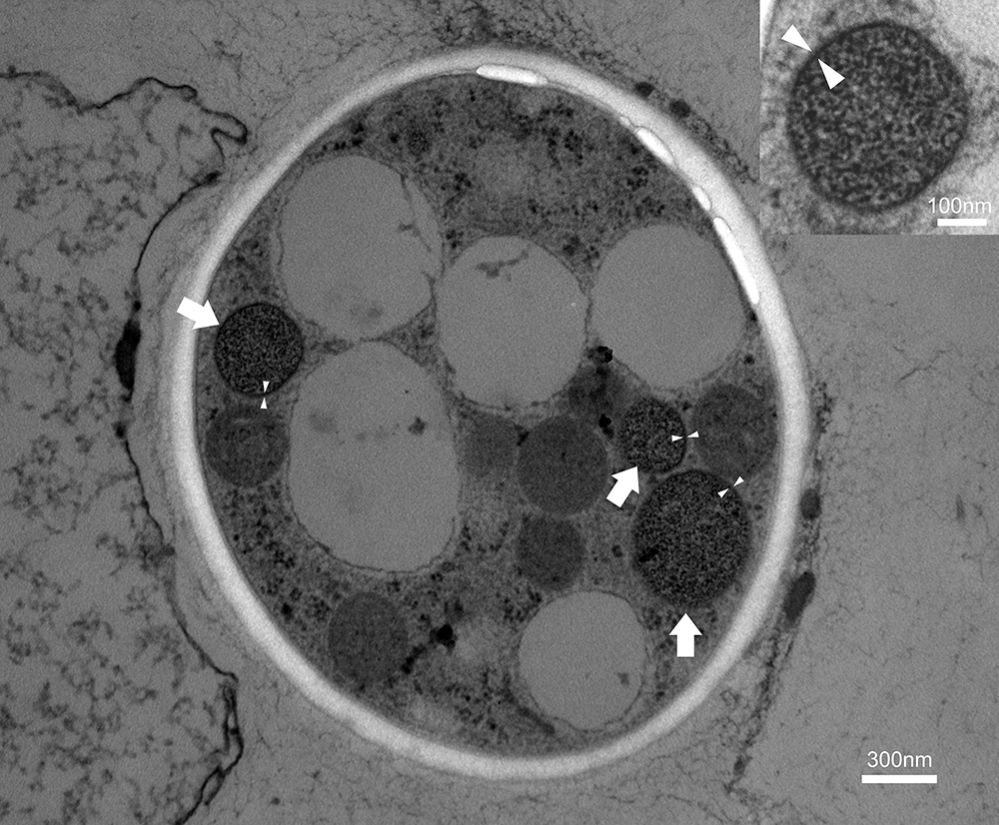
Transmission electron microscopic images of endobacteria in conidia of *Metarhizium bibionidarum* CBS 648.67. Endobacteria are indicated with arrows and cell walls with triangles.

To validate the molecular detection of endobacteria, TEM was used to identify the cytoplasmic locations of endobacteria within the conidia of *M. bibionidarum* CBS 648.67. Endobacteria directly embedded in the fungal cytoplasm were observed (Fig. 4) and were coccoid, similar to the endobacteria detected in *Esteya vermicola* (Wang *et al.*, 2017). These endobacteria also had a distinct cell wall structure in the outermost layer. The endobacteria observed in TEM images were 200–500‍ ‍nm in size (Fig. 4). The diameters of large endobacteria were approximately twice those of small endobacteria (Fig. 4), which indicated that large endobacteria were about to undergo cell division. This was corroborated by SEM results, as discussed below.

**Fig. 5.**
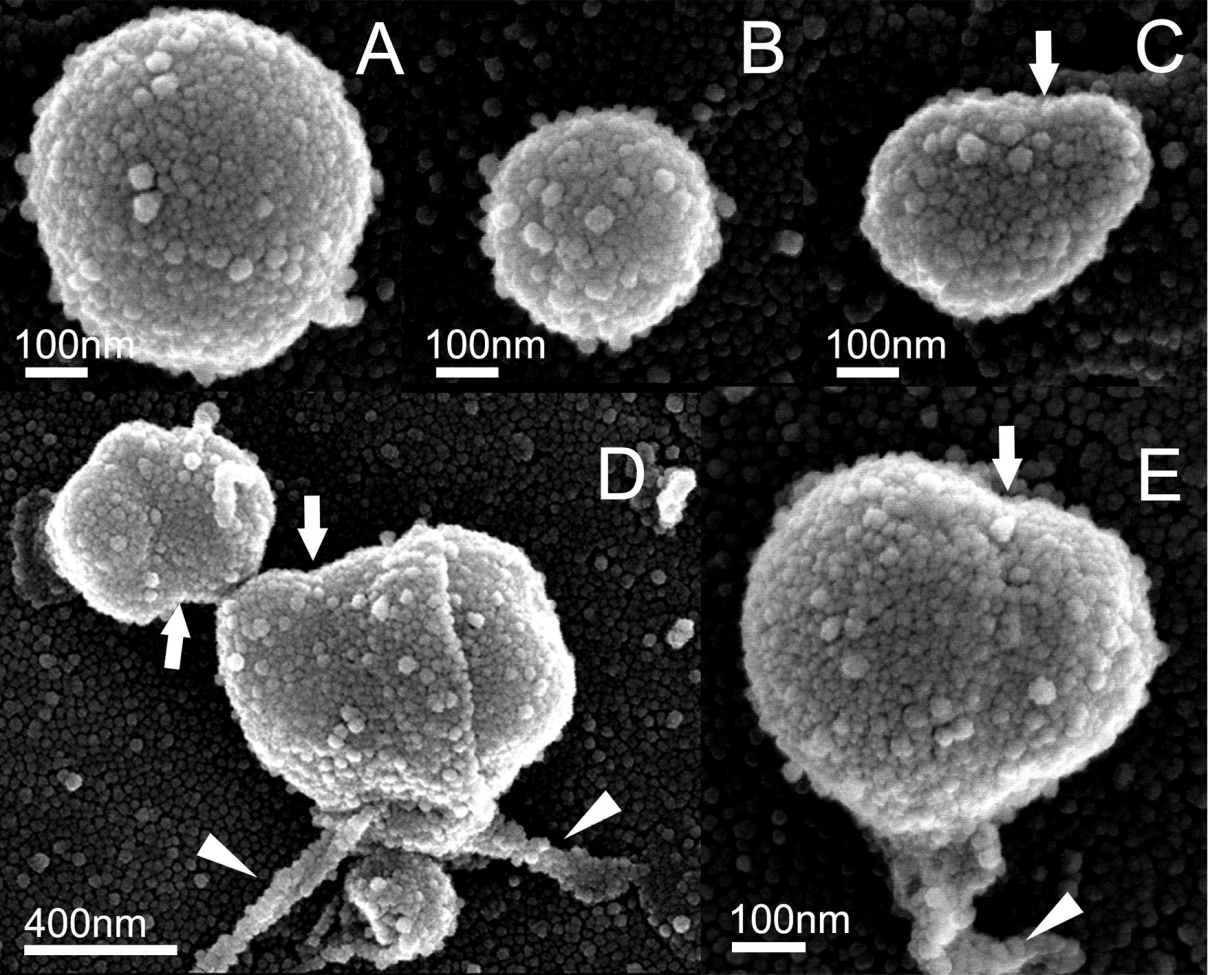
Scanning electron microscopic images of endobacteria from *Metarhizium bibionidarum* CBS 648.67. (C and E) The central constriction (arrows) suggests that endobacteria are engaged in cell division. (D and E) Fiber-like structures (indicated by white triangles).

Fractionated endobacteria were observed by SEM. All endobacteria were spherical (Fig. 5), and their diameters in the visual field were mostly less than 500‍ ‍nm. Fluorescence signals also revealed the presence of endobacteria in the isolated suspension (Fig. 3O), which indicated that the spherical structures were endobacteria. SEM images showed fiber-like structures firmly attached to the surfaces of endobacteria that were 20–40‍ ‍nm in diameter (Fig. 5D and E). These fiber-like structures were suspected to be microtubules (Schwan *et al.*, 2014; Wang *et al.*, 2017). The central constriction (Fig. 5C, D, and E) suggested that endobacteria propagated in the cytoplasm of fungi by cell division. The diameters of the dividing endobacteria were relatively large, at approximately 500‍ ‍nm.

## Discussion

A combination of ultrastructural, molecular, and phylogenetic ana­lyses demonstrated that the CBS 648.64 and CFCC 80795 strains of *Metarhizium* spp. hosted endobacteria in their conidia and hyphae. These results also showed that the presence of these bacteria was not due to exogenous bacterial contamination.

Endobacteria were abundant within fungal hyphae and conidia, as revealed by fluorescence staining using SYTO9 and FISH (Fig. 1 and 3). Their presence in both hyphae and conidia indicated that endobacteria inside CBS 648.67 were vertically transmitted from one generation to the next by sporulation (Fig. 3) and that they grew, multiplied, and migrated along with conidial germination.

The results of FISH, TEM, and SEM showed that the morphology of endobacteria within *M. bibionidarum* CBS 648.67 was coccoid. Endobacteria had cell walls with attached fibrous structural components that appeared to be microtubules rather than flagella. We speculated that similar to other fungal organelles, these endobacteria move inside the cell using microtubules (Dohner *et al.*, 2005; Abrahams and Hensel, 2006; Wang *et al.*, 2017). These endobacteria were smaller than the free-living close relative, *P. puraquae* (Gomila *et al.*, 2007), which may be an adaptation to the limited living space in the fungal host.

Based on the 16S rRNA ana­lysis, we concluded that endobacteria were closely related to *P. puraquae*, a free-living Gram-negative bacterium from the genus *Pelomonas* in the family *Comamonadaceae*. However, unlike *P. puraquae*, which is rod-shaped (Gomila *et al.*, 2007), the endobacteria found in the present study were coccoid. The genus *Pelomonas* was previously isolated from hemodialysis water, industrial water, oceans, and other oligotrophic environments (Gomila *et al.*, 2005; Gomila *et al.*, 2006). Some strains ascribed to *P. saccharophila* act as degraders of aromatic compounds (Stringfellow and Aitken, 1995), which are contaminants of pure and ultrapure water (Kulakov *et al.*, 2002; Gomila *et al.*, 2005). *P. puraquae* was shown to proficiently use various carbon sources (Gomila *et al.*, 2007). The presence of endobacteria from the genus *Pelomonas* in fungi isolated from plant seeds and leaves, as well as from grassland soils, was also confirmed in previous studies (Shaffer *et al.*, 2016; Muller *et al.*, 2021). Endobacteria from the genus *Pelomonas* were observed in *Pleurotus ostreatus* (Adamski and Pietr, 2019). However, the cultivability of these bacteria has not yet been demonstrated. Shaffer *et al.* (2018) observed the relatively powerful impact of endobacteria on shaping the effects of their fungal host Nectriaceae sp. on the viability of *Cecropia peltata* seeds. However, these effects were not solely attributed to *Pelomonas* endobacteria because of the simultaneous presence of multiple endobacteria in the fungus. Therefore, the role played by *P. puraquae* in fungi warrants further investigation.

Nitrogenase (*nifH*) and hydrogenase (*hoxG*) genes are relevant to the metabolism of the genus *Pelomonas*. The *nifH* gene is the most widely sequenced marker gene used to identify nitrogen-fixing bacteria and archaea, and the hydrogenase-related *hoxG* gene forms a heterodimeric, membrane-bound hydrogenase with *hoxK* (Kortlüke *et al.*, 1992; Gomila *et al.*, 2007; Gaby and Buckley, 2012). Amplification and sequencing results showed the absence of the target genes *nifH* and *hoxG* in endobacteria, which may be related to the inability of these bacteria to survive *in vitro* independently of the host fungus.

The cytoskeletal protein MreB is a bacterial ortholog of actin (Gaballah *et al.*, 2011). It is critically involved in cell shape (Figge *et al.*, 2004). Amplification by 11 primer pairs for the *mreB* gene and identification results showed that symbiotic bacteria lacked the *mreB* gene, which was different from free-living *Pelomonas*. The sequences amplified by the primer pair mreB-1/mreB-2 were highly similar to those of *M. brunneum* strain CP058938.1 (query cover 98%, identity 96.14%) in the NCBI database. However, FISH (Fig. 3G, H, I, J, K, L, M, and N) showed multiple fluorescence signals in single fungal cells, and the fluorescence signals of probe M were not localized to fungal nuclei, which suggested that the sequence was not present on the chromosome of the fungal host (if present, a single fluorescence signal will appear in each fungal nucleus). The fluorescence signals obtained by the hybridization of probe 16S (Fig. 3I and M) and probe M (Fig. 3J and N) highly overlapped, further indicating that the sequence exists in endobacteria, but not in their host. Bonfante *et al.* (2019) reported that endobacterial genomes contained a number of horizontally transferred genes of host fungal origin to complement the shortfall associated with gene reductions. Genome sequencing by Naito *et al.* (2015) provided evidence for horizontal gene transfer events, particularly fungal genes involved in posttranslational modifications (Naito *et al.*, 2015; Torres-Cortes *et al.*, 2015). Therefore, the sequences amplified using the primers mreB-1/mreB-2 may be transcripts that endobacteria carry from the fungal host. Sequences amplified using the ten designed primer pairs were not the *mreB* gene of *P. puraquae*. Previous studies reported that marked genomic reductions and accumulated mutations during the establishment of the symbiotic relationship with the host were important factors affecting morphological and functional changes in endobacteria (McCutcheon and Moran, 2011; Bonfante and Desiro, 2017; Deveau *et al.*, 2018; Bonfante *et al.*, 2019). Therefore, the lack of the *mreB* gene and the possible horizontal transfer of genes in the endobacteria of *Metarhizium* strains may change the endobacterial morphology from rod-shaped to spherical, which was consistent with previous findings on symbiotic bacteria in *E. vermicola* (Wang *et al.*, 2017).

Endobacteria play important roles in host morphology, host germination and growth, pathogen toxins, and nitrogen fixation, similar to mycorrhizal symbionts (Liu *et al.*, 2019). The degradation of a host’s lipid layer, the penetration of the host cuticle, and host colonization (overcoming host defense responses) are critical invasive steps by *Metarhizium* spp. (Schrank and Vainstein, 2010), and are closely related to its pathogenicity. In these steps, the production of enzymes that disrupt the integrity of the host and form toxins to overcome host defense responses result in a strong selective advantage for the pathogen. The endobacteria of *Rhizopus microsporus* have been identified as true producers of rhizoxin and ‘*mycotoxin*’ rhizonin (Partida-Martinez and Hertweck, 2005, 2007). Therefore, we hypothesized that similar relationships may exist between the pathogenic ability of *Metarhizium* on insect hosts and its endobacteria. Some bacteria of the genus *Pelomonas* exhibit not only viability in oligotrophic environments, but also the utilization of multiple carbon sources (Gomila *et al.*, 2007). Therefore, the endobacteria of *Metarhizium* may contribute to their insect host range, such as facilitating the utilization of nitrogen sources by fungi through the expansion of fungal nutrient assimilation capacities and ecological niches. These hypotheses warrant further experimental verification.

Endobacteria operate as multipliers of fungal genetic variability, providing diversity for natural selection (Bonfante *et al.*, 2019). The host ranges and virulence levels of *Metarhizium* strains vary (Zimmermann, 2007). Research on endobacteria will provide a more detailed understanding of the phylogeny of *Metarhizium*, insights into interactions with insects and their pathogenic mechanisms, and expand the application of *Metarhizium*.

## Citation

Ying, Y., Liu, C., He, R., Wang, R., and Qu, L. (2022) Detection and Identification of Novel Intracellular Bacteria Hosted in Strains CBS 648.67 and CFCC 80795 of Biocontrol Fungi *Metarhizium*. *Microbes Environ ***37**: ME21059.

https://doi.org/10.1264/jsme2.ME21059

## Supplementary Material

Supplementary Material 1

Supplementary Material 2 Figure S2 Z-stack of hyphae (probe 16S and DAPI)

Supplementary Material 3 Figure S3 Z-stack of growing conidia (probe 16S and DAPI)

Supplementary Material 4 Figure S4 Z-stack of hyphae (probe EUB338 mix and DAPI)

Supplementary Material 5 Figure S5 Z-stack of hyphae (probe EUB338 mix and DAPI)

Supplementary Material 6 Figure S6 Time series images (probe 16S)
