# Supplementary material for: Detection and Identification of Novel Intracellular Bacteria Hosted in Strains CBS 648.67 and CFCC 80795 of Biocontrol Fungi Metarhizium
Source: Microbes Environ. 2022 May 24;37(2):ME21059. doi: 10.1264/jsme2.ME21059 (PMC9530730; doi:10.1264/jsme2.ME21059)
Supplement: Supplementary file 1 — Supplementary Material 1 [file 37_21059_s1.pdf]

Supplemental materials:

Table S1 Sequence and reference sequence of primers used for amplification of *mreB* genes of *P. puraquae*

Figure S1 Gel image of PCR products. (A) PCR products of CFCC 80795 amplified with primers 19F and 407R; (B) PCR products of CBS 648.67 amplified with primers 19F and 407R; (C) PCR products of CFCC 80795 amplified with primers *hox*F1 and *hox*R1; (D) PCR products of CBS 648.67 amplified with primers *hox*F1 and *hox*R1.

Figure S2 Z-stack of hyphae (probe 16S and DAPI)

Figure S3 Z-stack of growing conidia (probe 16S and DAPI)

Figure S4 Z-stack of hyphae (probe EUB338 mix and DAPI)

Figure S5 Z-stack of hyphae (probe EUB338 mix and DAPI)

Figure S6 Time series images (probe 16S)

Table S1 Primer sequence designed and reference sequence accession used for amplification of *mreB* genes of *P. puraquae*

Primer	Sequence (5' to 3')	Reference sequence accession
MreB_LMDC_F1	ATGTTCGCCTCCCTGCGT	LMDC01000012.1
MreB_LMDC_R1	TTATTCAGACGTGAAGATCGAACC	
MreB_LMDC_F2	ATGTTCGCCTCCCTGCGTC	LMDC01000012.1
MreB_LMDC_R2	TTATTCAGACGTGAAGATCGA	
MreB_LMDK_F1	ATGTTCGCCTCCCTGCGC	LMDK01000001.1
MreB_LMDK_R1	TCACTCCGACGTGAAGAT	
MreB_LMDK_F2	ATGTTCGCCTCCCTGCGCCGCTAT	LMDK01000001.1
MreB_LMDK_R2	TCACTCCGACGTGAAGATCGAGCC	
MreB_LMDU_F1	ATGTTCGCCTCCCTGCGCCGTTATTTC	LMDU01000011.1
MreB_LMDU_R1	TTATTCCGAAGTGAAGATGGAGCCCAG	
MreB_LMFP_F1	ATGTTCGCCTCCCTGCGC	LMFP01000013.1
MreB_LMFP_R1	TTATTCCGAAGTGAAGATGGA	
MreB_LMHE_F1	ATGTTCGCCTCCCTGCGCCGTTATTTC	LMHE01000011.1
MreB_LMHE_R1	TTATTCCGAAGTGAAGATGGAGCCCAG	
MreB_SMBU_F1	ATGTTCGCCTCCCTGCGC	SMBU01000028.1
MreB_SMBU_R1	TTATTCGGAGGTGAAGATGC	
MreB_SMBU_F2	ATGTTCGCCTCCCTGCGCCGCTAT	SMBU01000028.1
MreB_SMBU_R2	TTATTCGGAGGTGAAGATGCTGCCCA	
MreB_LMDU_LMFP_LMHE_F2	ATGTTCGCCTCCCTGCGCCGTTAT	LMDU01000011.1
MreB_LMDU_LMFP_LMHE_R2	TTATTCCGAAGTGAAGATGGAGCC	LMFP01000013.1
		LMHE01000011.1

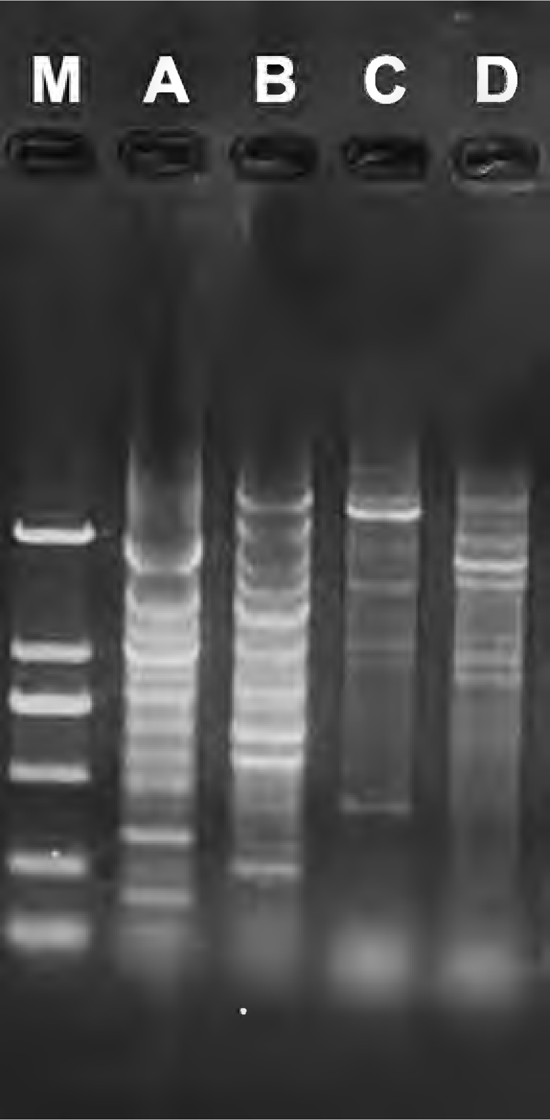

M
A
B
C
D
